# AI-powered topic modeling: comparing LDA and BERTopic in analyzing opioid-related cardiovascular risks in women

**DOI:** 10.3389/ebm.2025.10389

**Published:** 2025-02-28

**Authors:** Li Ma, Ru Chen, Weigong Ge, Paul Rogers, Beverly Lyn-Cook, Huixiao Hong, Weida Tong, Ningning Wu, Wen Zou

**Affiliations:** ^1^ Division of Bioinformatics and Biostatistics, National Center for Toxicological Research, U.S. Food and Drug Administration, Jefferson, AR, United States; ^2^ Department of Information Science, University of Arkansas at Little Rock, Little Rock, AR, United States; ^3^ Office of New Drug, Center for Drug Evaluation and Research, U.S. Food and Drug Administration, Silver Spring, MD, United States; ^4^ Division of Biochemical Toxicology, National Center for Toxicological Research, U.S. Food and Drug Administration, Jefferson, AR, United States

**Keywords:** AI, BERTopic, topic modeling, opioid, cardiovascular risks

## Abstract

Topic modeling is a crucial technique in natural language processing (NLP), enabling the extraction of latent themes from large text corpora. Traditional topic modeling, such as Latent Dirichlet Allocation (LDA), faces limitations in capturing the semantic relationships in the text document although it has been widely applied in text mining. BERTopic, created in 2022, leveraged advances in deep learning and can capture the contextual relationships between words. In this work, we integrated Artificial Intelligence (AI) modules to LDA and BERTopic and provided a comprehensive comparison on the analysis of prescription opioid-related cardiovascular risks in women. Opioid use can increase the risk of cardiovascular problems in women such as arrhythmia, hypotension etc. 1,837 abstracts were retrieved and downloaded from PubMed as of April 2024 using three Medical Subject Headings (MeSH) words: “opioid,” “cardiovascular,” and “women.” Machine Learning of Language Toolkit (MALLET) was employed for the implementation of LDA. BioBERT was used for document embedding in BERTopic. Eighteen was selected as the optimal topic number for MALLET and 23 for BERTopic. ChatGPT-4-Turbo was integrated to interpret and compare the results. The short descriptions created by ChatGPT for each topic from LDA and BERTopic were highly correlated, and the performance accuracies of LDA and BERTopic were similar as determined by expert manual reviews of the abstracts grouped by their predominant topics. The results of the t-SNE (t-distributed Stochastic Neighbor Embedding) plots showed that the clusters created from BERTopic were more compact and well-separated, representing improved coherence and distinctiveness between the topics. Our findings indicated that AI algorithms could augment both traditional and contemporary topic modeling techniques. In addition, BERTopic has the connection port for ChatGPT-4-Turbo or other large language models in its algorithm for automatic interpretation, while with LDA interpretation must be manually, and needs special procedures for data pre-processing and stop words exclusion. Therefore, while LDA remains valuable for large-scale text analysis with resource constraints, AI-assisted BERTopic offers significant advantages in providing the enhanced interpretability and the improved semantic coherence for extracting valuable insights from textual data.

## Impact statement

This study provides a comparative analysis of LDA and BERTopic in the context of AI-driven topic modeling to analyze opioid-related cardiovascular risks in women. While both methods were capable of effectively identifying topics within text corpora, our findings reveal that BERTopic offers obvious advantage due to its seamless integration with AI techniques and improved semantic coherence in text documents. In addition, it uncovered themes related to opioid-associated health risks and outcomes in specialized patient groups, including pregnant patients and those undergoing coronary surgery. BERTopic’s ability to automatically incorporate contextual information through transformer-based models makes it particularly well-suited for AI generation tasks, where adaptability and precision are critical. In comparison, LDA, although performing well, requires data pre-processing and manual adjustments to achieve similar levels of AI integration. These results underscore the potential importance of AI integration into topic modeling techniques in the analysis of large-scale biomedical text data to achieve more accurate and meaningful insights. This integration not only enhances the precision of topic modeling but also accelerates the modeling and output interpretation, potentially empowering researchers and practitioners with varying levels of expertise to derive valuable insights from unstructured text data.

## Introduction

The opioid epidemic has become a serious national crisis in the United States, with far-reaching consequences across various populations [1]. In 2023, nearly 8.6 million Americans 12 years and older reported misusing prescription opioids and over 5 million reported a prescription use disorder in the past year [2]. It was reported that approximately 294,000 people died from overdoses involving prescription opioids from 1999 to 2022 [3]. Healthcare systems bear substantial costs due to increased hospitalizations and emergency department visits associated with opioid overdoses [4]. Women have seen a marked rise in opioid-related issues, for example, since 1999 deaths from prescription opioid overdoses increased 642% among women compared with a 439% increase among men [5]. Among these, women face unique cardiovascular risks associated with opioid use, necessitating targeted research to understand these complex relationships [6].

Natural language processing (NLP) has become an essential tool for extracting meaningful insights from vast amounts of biomedical literature, such as PubMed abstracts. Topic modeling has emerged as a foundational technique within the domain of NLP and text mining, providing an essential methodology for extracting insightful patterns from extensive and intricate text datasets. As an unsupervised machine learning approach, topic modeling discerns latent themes or topics within a corpus of documents, thereby facilitating the systematic organization, comprehension, and extraction of meaningful patterns from vast amounts of unstructured text data, where manual analysis is both impractical and infeasible [7–10].

The utilization of topic modeling extends across various fields and applications. It is broadly implemented in document classification and organization, text summarization, customer feedback analysis, sentiment analysis, trend analysis, bioinformatics analysis, and even biological and biomedical research [11–18]. Traditional topic modeling methods such as Latent Dirichlet Allocation (LDA) have established a robust framework for understanding and organizing unstructured text data. While these conventional techniques have proven effective across various contexts, they face evident limitations in capturing the intricate semantic relationships within increasingly complex and voluminous datasets [19], such as medical literature. Additionally, the outputs produced by these traditional methods often pose interpretability challenges, particularly for individuals who lack domain-specific expertise [11, 13, 20, 21].

BERTopic was developed by Grootendorst in 2022 by leveraging advances in deep learning, especially those in transformer-based models such as Bidirectional Encoder Representations from Transformers (BERT) which excels in generating deep contextualized word embeddings by considering the full context of words in a sentence, as read from both the left and the right [22]. While LDA (which employs the “bag-of-words” method) and TF-IDF etc. ignore the order and context of words [23], BERTopic uses BERT embeddings which can capture the contextual relationships between words [24]. BERTopic consists of four major modularized steps: document embedding, dimensionality reduction, clustering, and topic representation [22]. These steps are independent from each other. Each of these steps can be modified or replaced without affecting the others. This allows users to customize and tailor the topic modeling processing by integrating different algorithms or techniques at different stages of the pipeline. [25]. Therefore, BERTopic has an important advantage in its modularity. This flexibility is able to extend integrating an Artificial Intelligence (AI) module for enhancing result interpretability.

**FIGURE 1:**
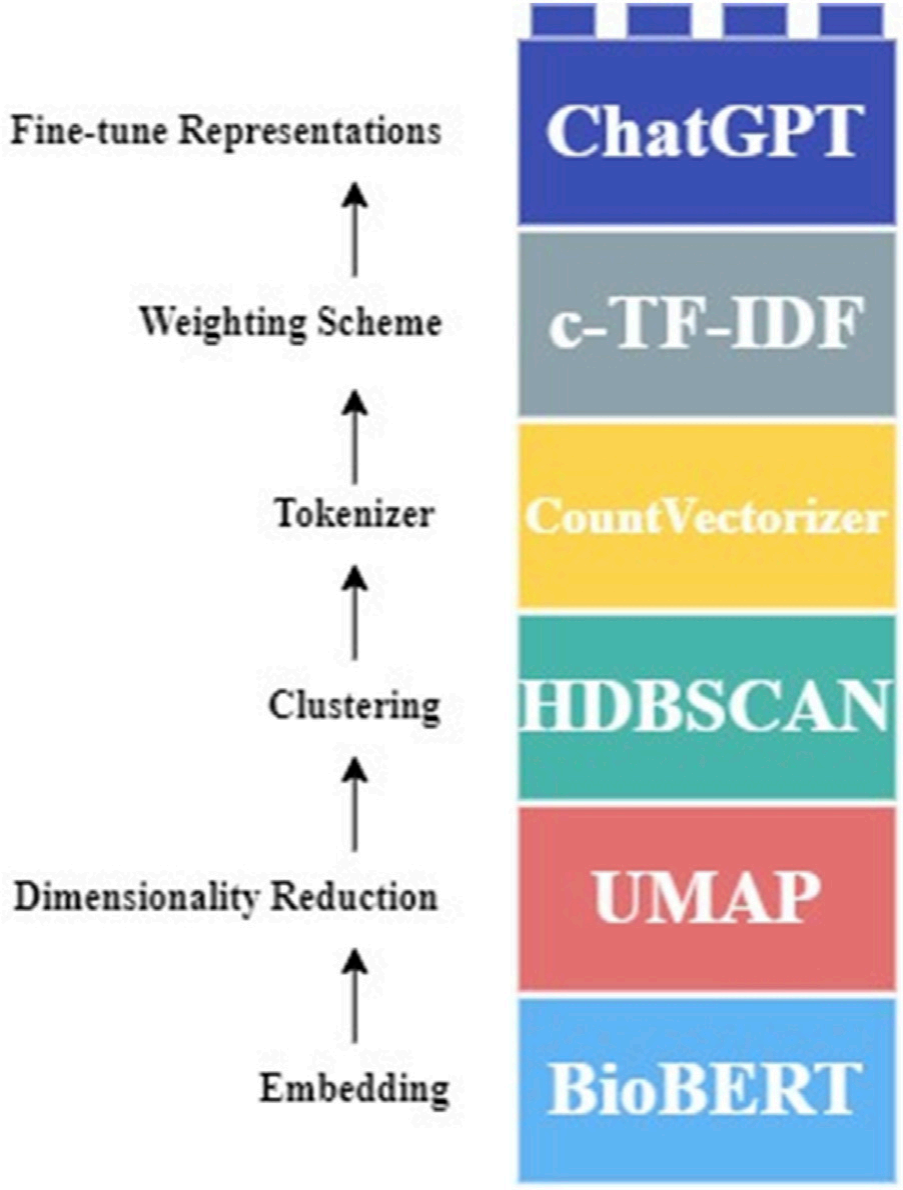
Customized BERTopic pipeline used for this study. The abstracts were first embedded using BioBERT. Then UMAP was employed for dimensionality reduction, followed by HDBSCAN for clustering. CountVectorizer was then used for tokenization and handling the stop words. c -TF-IDF was applied for a weighting scheme. Lastly, fine-tuning representations was achieved with ChatGPT, enhancing the interpretability and quality of the topic modeling outputs.

Multiple studies have applied topic modeling to analyze text data from social media platforms, electronic health records, and adverse event reporting systems to understand patterns of opioid misuse and its societal impact. For instance, we have applied LDA to perform text mining on prescription opioids-related literatures in PubMed to capture the research themes and to explore the prevalent topic dynamics in the literatures [6]. LDA has also been utilized to examine X (previously Twitter data) for trends in public sentiment and discourse around opioid use, highlighting a range of topics including how opioids are administered, opioid use affecting life and withdrawal symptoms due to trying to quit opioids [26]. Although BERTopic modeling is a relatively new algorithm, it has been applied in the text mining in many fields [27, 28]. These approaches underscore the potential of topic modeling to inform policy, improve surveillance systems, and enhance targeted interventions addressing the opioid crisis.

AI has significantly evolved over the past few years and has found applications in an increasing number of fields. This study delves into the innovative integration of AI to aid in the interpretation of results derived from traditional topic modeling approaches like LDA and contemporary methods such as BERTopic. To comprehensively compare the performance of AI-integrated LDA and BERTopic, we used a curated dataset of biomedical abstracts retrieved from PubMed for prescription opioids-related cardiovascular issues in women. By applying these two AI-integrated models to this specific dataset, we aimed to evaluate their effectiveness and accuracies in uncovering the intricate themes within the literature and their ability to handle the complexities inherent in biomedical texts. The comparison focused on the coherence, contextual relevance, and ease of use of each model, providing insights into their respective strengths and limitations.

## Materials and methods

### Data collection and preprocessing

The PubMed abstract collection was pursued in April 2024 by utilizing the PubMed file retrieval tool easyPubMed v2.13. All the available abstracts which were published before April 15th, 2024, were obtained by searching PubMed with three mesh words: “opioid”, “cardiovascular”, and “women” based on the following criteria: a). language: only English-language abstracts were included; b). availability: abstracts that are freely accessible and available in full text; and c). species: the search scope was restricted to human related research. The search query was set up as “cardiovascular AND opioid AND humans [mh] AND english [la] AND [(women) OR (female)] NOT exclude preprints [Publication Type]”.

Datasets collected directly from PubMed may contain noisy information that can compromise the relevance of the results [18]. The curated abstract dataset was preprocessed firstly by removing numbers, punctuations, special characters, html tags and URLs from the dataset using sed (stream editor in Linux). Next, the Stanford NLP tool Stanza [29] was employed to lemmatize the pre-processed text data to remove inflectional endings and to convert a word back to its root form (e.g., running to run). Stop words were excluded by the *remove-stopwords* function in MALLET (Machine Learning for Language Toolkit) [30].

Unlike LDA, BERTopic does not need data preprocessing and uses the original sentences in the curated dataset as the original structures of the texts play vital roles for BERTopic’s transformer models.

### Implementation of topic models

#### Model 1: ChatGPT powered LDA

The latest version, MALLET v2.0.8, was installed and used under openJDK 11.0.22. to implement LDA. MALLET is a Java-based package designed for statistical natural language processing, document classification, topic modeling, information extraction, and other machine learning applications regarding textual data [30].

As a crucial step in topic modeling, determining the optimal number of topics for a LDA model can significantly influence the quality and interpretability of the results [31]. It is often done through time-consuming trial-and-error or using perplexity-based methods which may not always yield stable results. In this study, the Rate of Perplexity Change (RPC)-based approach [32] was adopted to determine the optimal number of topics in LDA. This method aims to find the first change point in the RPC which implies the most appropriate number of topics for a given dataset.

MALLET was then re-run on the same dataset with all the same settings except changing the parameter for topic number to the most fitting one. The top 100 words of each topic along with their corresponding probabilities and the abstracts clustered in the topic were uploaded to ChatGPT-4-Turbo to generate a one-sentence description for each topic.

#### Model 2: BERTopic

As shown in Figure 1, BioBERT was selected for the document embedding as the first step [33]. For dimensionality reduction and clustering, the default algorithms of Uniform Manifold Approximation & Projection (UMAP) [34] and Hierarchical Density-Based Spatial Clustering of Applications with Noise (HDBSCAN) [35] were used, respectively. Although not necessary to remove stop words from the data, CountVectorizer from the sklearn package [36] was utilized to handle the stop words. ChatGPT-4-Turbo was employed as the last step.

### Performance comparison

#### Accuracy of relevance by manual expert review

To validate the accuracies of the outcomes from the two AI-integrated topic modeling approaches, we randomly picked one topic from each group of topics generated by the two approaches. The abstracts which were clustered into the two topics were read by domain experts to manually evaluate if the abstracts properly aligned with the respective topics.

#### Abstract clusters by visualization analysis

Each abstract was labeled with the topics and their corresponding probabilities, derived from LDA and BERTopic, respectively. t-SNE (t-distributed Stochastic Neighbor Embedding) [37] was utilized to reduce the 18-dimensional (LDA MALLET)/21-dimensional (BERTopic) topic probabilities to 3 dimensions prior to clustering the abstracts. Each cluster was then evaluated to see if the abstracts in a cluster had the same topic number.

#### Coherence comparison

In addition to comparing the topic results manually, UMass coherence score [38] was utilized to evaluate the performance of the two methods, BERTopic and LDA (MALLET). UMass coherence scores for each topic were calculated by using the Python package gensim v3.8.3 [39]. An acceptable coherence score should be a value between −14 and 14 according to the genism documentation [40].

## Results and discussion

**FIGURE 2:**
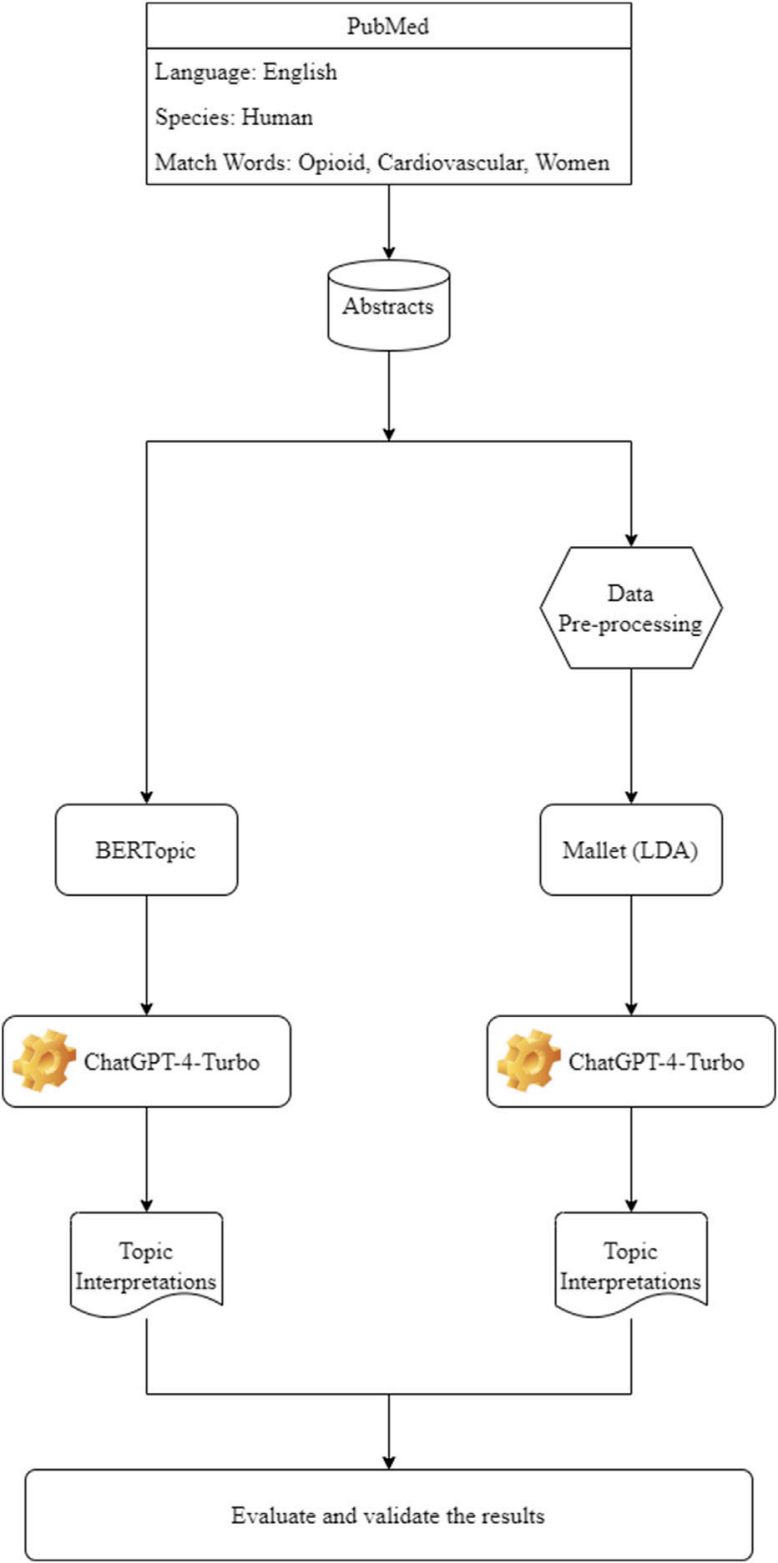
Research workflow.

Currently, the development of AI has raised an interesting question on how to integrate the AI/large language models with the traditional NLP to enhance the capabilities of language processing systems, enabling more accurate, context-aware, and sophisticated analysis of text data. Traditional NLP methods often rely on rule-based approaches and statistical models, while AI, particularly through machine learning and deep learning, brings a more dynamic and context-aware understanding of language. In this study, we integrated ChatGPT4-Turbo to traditional MALLET-based LDA and BioBERT-embedded BERTopic and underscore the transformative potential of AI in enhancing topic modeling techniques. A dataset of PubMed abstracts focused on opioid-related cardiovascular risks in women was used as a case study. The objective was to compare and evaluate the performance of these two topic modeling techniques leveraged by the advances of AI algorithms in uncovering meaningful themes within a specialized biomedical dataset. The proposed approach can be applied to any topic modeling algorithm coupled with an AI system in the downstream pipeline through either manually implementation or automatic connection with programming language interface. Figure 2 illustrates the workflow for this study. 1,837 abstracts were retrieved and downloaded from PubMed through April 2024 using three MeSH words: “opioid,” “cardiovascular,” and “women,” followed by processing by MALLET topic modeling or BERTopic. The outputs of the two topic modeling approaches were compared from several aspects following integration with ChatGPT-4-Turbo manually or automatically.

### Ease of large language model integration and use

**FIGURE 3:**
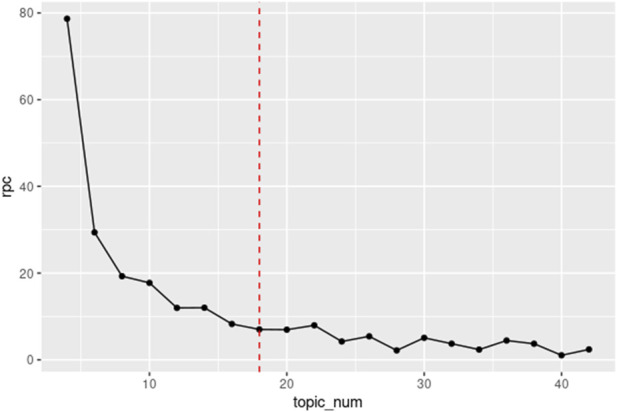
The Rate of Perplexity Change (RPC) Plot. Illustration of the RPC as a function of the number of topics (topic_num) for LDA modeling. The y-axis represents the RPC, while the x-axis indicates the number of topics.

As shown in Figure 2, the integration of ChatGPT-4-Turbo or other large languages with LDA required additional steps, including data pre-processing, tuning hyperparameters and manually uploading the LDA outputs to ChatGPT-4-Turbo for refining the generated topics. The RPC-based method was applied to calculate the rate of change in statistical perplexity, and using this approach, 18 was identified as the optimal topic number for the LDA topic modeling (Figure 3). These additional data processing steps were time-consuming and labor-intensive, and required a deep understanding of both the model and the dataset to achieve optimal results, especially when dealing with a large and complicated dataset.

BERTopic provided a simpler and streamlined experience. Since BERTopic technically determines the optimal topic number and the other parameters by the algorithm itself, it is not necessary to spend extra time tuning parameters. Consequently, BERTopic generated 21 topics from this dataset. The integration of ChatGPT-4-Turbo is an inherent part of the BERTopic, therefore, the ease of use and automatic handling of context made BERTopic more accessible and efficient, reducing the need for extensive manual intervention.

### Topic coherence and interpretability

Table 1 shows the output topics from AI-integrated LDA model (18 topics) and BERTopic model (21 topics), that were subsequently interpreted by ChatGPT-4-Turbo. The MALLET-based LDA model generated 18 topics across the dataset (Table 1). The topics generated by LDA were coherent and interpretable, for example, Topic 1, explored the “interactions and effects of opioids on cardiovascular and autonomic nervous system responses,” with a broad focus on “blood pressure, heart rate, and sympathetic activity.” The manual integration of AI techniques improved coherence to an extent and lowered dependency on expertise. While relevant themes were captured, the interpretations from ChatGPT-4-Turbo seemed to lack contextual specificity, potentially due to the indirect manual integration of the LDA outputs into ChatGPT-4-Turbo.

**TABLE 1:** The topics generated by ChatGPT-integrated BERTopic and LDA.

BERTopic	
Topic	Topic Labels by ChatGPT-4-Turbo
1	Effects of Remifentanil on Cardiovascular Response during Anesthesia Induction and Intubation
2	Cardiovascular Effects of Various Anesthetics in Coronary Surgery
3	Anesthesia Management in Pregnant Patients with Cardiac and Pulmonary Complications
4	Opioid Use and Associated Health Aspects in Various Patient Populations
5	Opioid Use and Associated Health Aspects in Various Patient Populations
6	Postoperative Pain Management in Cardiovascular Surgeries
7	Comparative Analyses of Opioid and Sedative Efficacy in Postoperative and Intensive Care Settings
8	Methadone Use and Associated Cardiovascular Risks
9	Opioid and Drug Use Disorders in Hospitalized Patients
10	Opioid-Associated Risks and Mortality in Medical Settings
11	Opioids and Health Outcomes in Specific Patient Populations
12	Cardiovascular Effects and Analgesia in Anesthesia Procedures
13	Opioid Use, Migraine Management, and Associated Health Outcomes
14	Opioid Effects and Management in Postoperative and Cardiac Care
15	Opioid and Opium Use Impact on Health and Mortality in Iran
16	Cardiovascular Effects of Anesthesia in Surgical Patients
17	Opioid and Anesthetic Effects on Cardiovascular and Hemostatic Responses in Clinical Settings
18	Management of Pain and Opioid Use in Clinical Settings
19	Effects of Salvinorin and Related Compounds on Opioid Receptors and Vascular Responses in Humans and Animal Models
20	Opioid Receptor Activation and Its Effects on Cellular and Physiological Responses
21	Drug Use and Health Outcomes in Special Populations

MALLET	
Topic	Topic Labels by ChatGPT-4-Turbo
1	Topic_1 primarily explores the interactions and effects of opioids on cardiovascular and autonomic nervous system responses, particularly focusing on changes in blood pressure, heart rate, and sympathetic activity during various medical procedures and conditions
2	The topic primarily focuses on the association between opioid use and various health outcomes, particularly cardiovascular diseases, in different populations
3	This topic primarily focuses on the medical complications and management associated with intravenous drug use, particularly in relation to infective endocarditis, vascular injuries, and the administration of anesthesia and analgesia in surgical settings
4	The topic primarily focuses on the clinical efficacy, safety, and comparative analysis of various pharmacological treatments, including opioids, NSAIDs, and other analgesics, in managing pain and related symptoms across different medical conditions and patient populations
5	The topic focuses on the effects and management of analgesia and anesthesia during labor and delivery, examining their impact on maternal and neonatal outcomes, including fetal heart rate patterns and drug exposure
6	The topic focuses on the comparison and evaluation of various sedative and analgesic drugs, their combinations, and administration techniques in managing pain and sedation during medical procedures, emphasizing their effects on cardiovascular and respiratory systems, efficacy, safety, and patient recovery outcomes
7	This topic focuses on the clinical outcomes, interventions, and trials related to acute myocardial infarction (AMI), including the use of various medications and their effects on patient recovery and complications
8	Topic 8 focuses on the comparison and evaluation of different surgical and anesthetic techniques in managing postoperative outcomes, pain, and complications in various medical procedures, emphasizing the effectiveness, safety, and impact on recovery times and hospital stays
9	This topic focuses on the study of opioid effects and interactions, particularly in relation to pain, stress responses, and various physiological and behavioral outcomes
10	The effects of various anesthetic agents and techniques on cardiovascular and hemodynamic responses during surgical procedures
11	Topic_11 focuses on the comparison and evaluation of different analgesic techniques and medications for postoperative pain management, particularly in relation to their efficacy, side effects, and impact on recovery in various surgical settings
12	The topic primarily focuses on the health impacts, particularly cardiovascular and pulmonary issues, associated with substance abuse and overdose, including the effects of various drugs like opioids, cocaine, and methadone on the human body
13	Topic 13 focuses on the effects and management of anesthesia, particularly in relation to cardiovascular stability, respiratory effects, and recovery times, with a specific emphasis on various anesthetic agents and techniques used during surgical procedures
14	Pharmacokinetics and pharmacodynamics of opioids and other drugs, focusing on their distribution, clearance, and effects within the body, particularly in relation to the blood-brain barrier and various bodily fluids
15	Polypharmacy and drug interactions in elderly patients, focusing on adverse drug reactions, prescribing patterns, and the impact of specific medications on health outcomes
16	Topic_16 focuses on the management and outcomes of opioid use in various medical settings, including treatment programs and emergency interventions, with an emphasis on the impact of opioid-related complications and mortality rates
17	Topic 17 primarily explores the cardiovascular effects and electrophysiological properties of various substances, including opioids and anesthetics, on the human heart, particularly focusing on QT interval prolongation, heart rate, and blood pressure responses
18	This topic involves the study of opioid receptors and their interactions with various peptides and antagonists, focusing on their effects on cardiovascular and neuroendocrine responses, as well as their potential therapeutic applications in conditions like pain management, shock, and cardiac function

Comparatively, the abstracts in the same dataset were first embedded using BioBERT (Figure 2). The use of transformer-based contextual embeddings enabled BERTopic to distinguish between closely related concepts, such as different cardiovascular effects of various anesthetic agents and opioid use during specific medical procedures. Consequently, BERTopic generated 21 distinct topics, with clear, contextually rich labels (Table 1). For instance, Topic 1 focused on the “Effects of Remifentanil on Cardiovascular Response during Anesthesia Induction and Intubation,” while Topic 12 highlighted “Cardiovascular Effects and Analgesia in Anesthesia Procedures.” These topics demonstrated BERTopic’s ability to generate specific and clinically relevant themes directly tied to the nuances of medical procedures and conditions. In addition, BERTopic excelled in maintaining contextual relevance. For instance, BERTopic differentiated between opioid-induced complications in various medical settings, such as postoperative care and labor (Topic 6, 7, 14), without the need for extensive manual adjustments.

We also calculated the UMass coherence scores to evaluate the performance of the topic modeling (Table 2). The calculation of the UMass coherence score provided a quantitative measure of topic quality based on word co-occurrence patterns within the dataset. More negative values represent that the words rarely co-occur, while values closer to zero indicate a higher tendency for words to co-occur [38, 41]. The UMass coherence scores of the 18 topics generated by LDA MALLET fall within the interval of −3.1 and −1 (Table 2a); and the coherence scores of the 21 topics from BERTtopic fall within the interval of −1.1 and 0 (Table 2b). All the coherence scores were between −14 and 14, and therefore considered as reasonable according to the genism documentation [40]. Comparatively, higher coherence scores for the topics from BERTopic (Table 2b) indicated that the words within a topic are more semantically related, reflecting better topic interpretability for the dataset.

**TABLE 2:** Coherence scores of the topics generated by LDA (MALLET) (a) and BERTopic (b).

(a)	
Topic	UMass coherence score
1	−1.5384
2	−2.0769
3	−1.4679
4	−1.9958
5	−2.1126
6	−1.8969
7	−2.1451
8	−1.9566
9	−2.0757
10	−1.7339
11	−3.0060
12	−2.2331
13	−1.3128
14	−1.9189
15	−1.5609
16	−1.7165
17	−1.8042
18	−1.3799

(b)	
Topic	UMass coherence score
1	−0.1023
2	−0.0798
3	−0.0593
4	−0.0531
5	−0.2831
6	−0.4271
7	−0.1564
8	−0.2707
9	−0.0783
10	−0.1169
11	−0.2800
12	−0.0983
13	−0.1544
14	−0.4083
15	−0.1719
16	−1.0587
17	−0.6269
18	−0.6592
19	−0.7001
20	−0.9951
21	−0.1447

### Relative accuracy

Topic 7 generated by MALLET-based LDA, and Topic 10 generated by BERTopic were randomly selected and expert reviews were conducted to assess the accuracy of topic relevancies of the clustered abstracts. Supplementary Figure S1A, S1B are the word clouds generated from Topic 7 using LDA and from Topic 10 using BERTopic, respectively. Topic 7 generated using LDA is interpreted as “focuses on the clinical outcomes, interventions, and trials related to acute myocardial infarction (AMI), including the use of various medications and their effects on patient recovery and complications” (Table 1). Results from two experts’ reviews revealed that 44 out of 49 (89.8%) abstracts were relevant to this topic (Supplementary Table S1A). Comparatively, Topic 10 from BERTopic was interpreted as “Opioid-Associated Risks and Mortality in Medical Settings” (Table 1), and the experts’ reviews revealed that 45 out of 53 (84.9%) abstracts were relevant to the topic (Supplementary Table S1B). The reviews also revealed that both models produced topics with similar levels of accuracy in terms of relevance to the opioid-related cardiovascular issues present in the dataset. The overall alignment with expert-identified themes was consistent across both approaches.

Although the two topics were randomly selected for both topic modeling approaches, the relatively lower level of accuracy of relevance from the BERTopic-generated topic might be due to the insufficient tuning of the parameters after embedding with BioBERT. In this study, we adopted the default setting provided by the developer, and ignored the fact that the hyperparameters, such as n_components, n_neighbors of UMAP, and the parameter min_cluster_size of HDBSCAN, may directly affect the decision for the number of topics after embedding, thereby affecting the topic outputs.

### Specificity by visualization analysis

**FIGURE 4:**
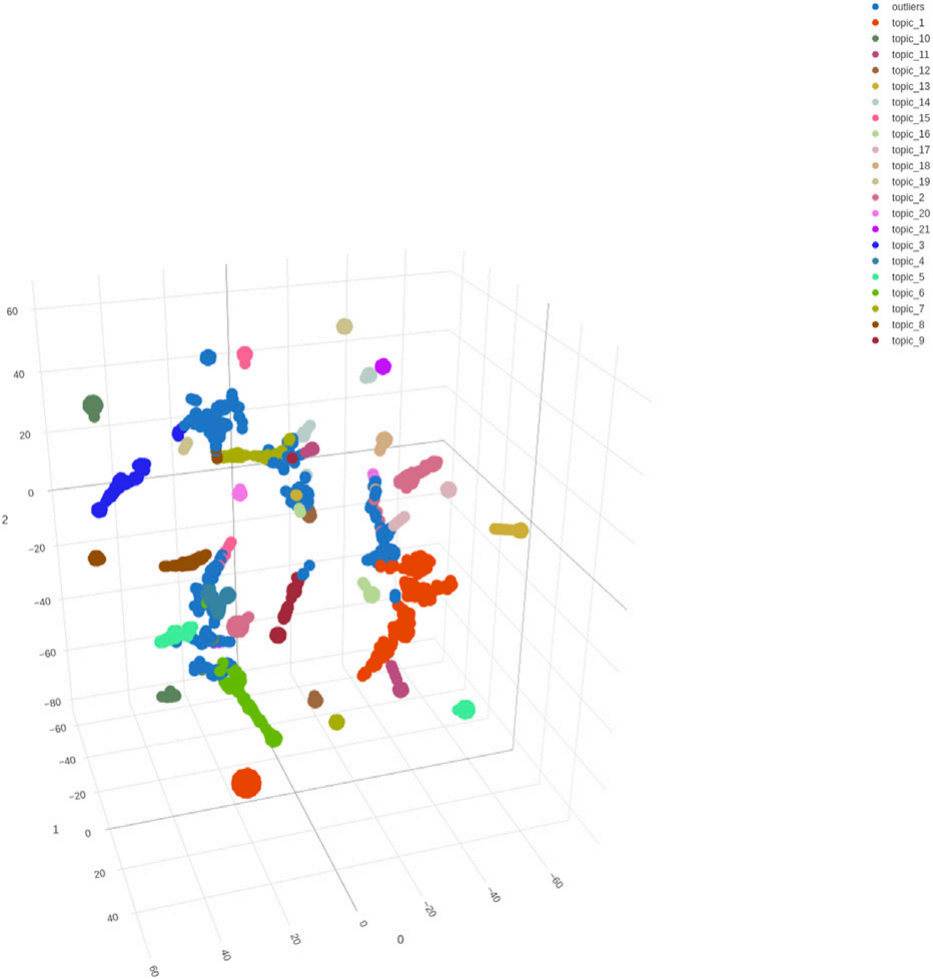
A 3D scatter plot visualization of the distribution of abstracts based on the topics identified by the BERTopic model. Each point represents an abstract and the color represents the abstracts assigned topic (Table 1). The axes represent the reduced dimensions obtained through t-SNE, which projects the high-dimensional topic space into three dimensions for better visualization.

**FIGURE 5:**
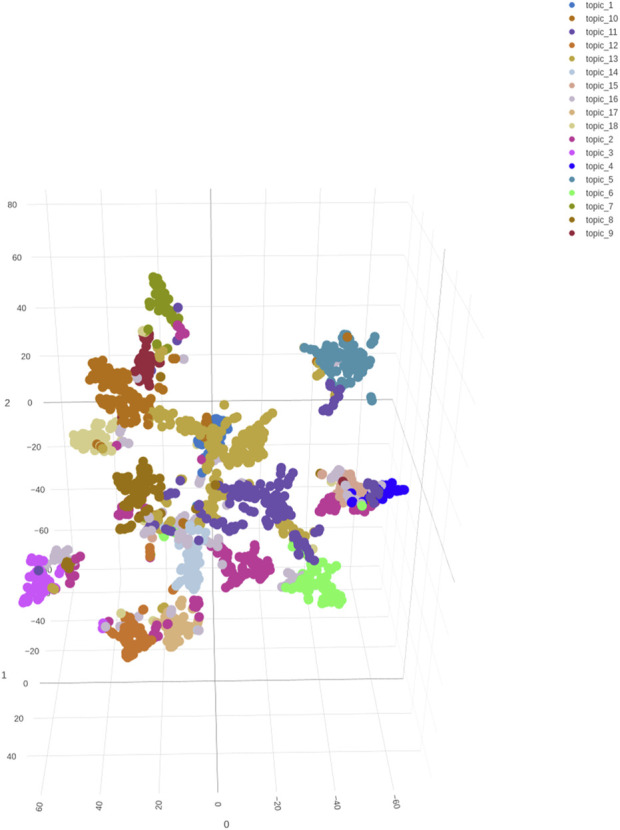
A 3D scatter plot visualization of the distribution of abstracts based on the topics identified by the MALLET-based LDA model. Each point represents an abstract and the color represents the abstracts assigned topic. The axes represent the reduced dimensions obtained through t-SNE, which projects the high-dimensional topic space into three dimensions for better visualization.

Hypothetically, each abstract is represented as a point in an 18-dimensional (LDA)/21-dimensional (BERTopic) space where each coordinate represents the probability that a corresponding topic occurring in the document. The high dimensionality of the data usually poses challenges for visualization. As such, t-SNE [37] was used to reduce the 18-dimensional/21-dimensional topic probabilities to 3 dimensions, respectively. This transformation provided an intuitive visualization of abstract distribution by plotting the data in 3-dimensional space. Figures 4, 5 show the distributions of abstracts labeled by BERTopic and Mallet, respectively. Each point represents an abstract, and each color represents a different topic to which the abstract was clustered. The 3D scatter plot provides a powerful tool for visually assessing the performance of the LDA and BERTopic models and understanding the structure of the topics within the dataset. Comparing the separation of clusters in both plots, BERTopic (Figure 4) shows a larger distance between the clusters and more distinct separation between the clusters as compared to LDA (Figure 5), which suggests better topic coherence and differentiation. Meanwhile, the LDA scatter plot (Figure 5) reveals more overlapping clusters than the BERTopic scatter plot (Figure 4), indicating that LDA topics are less distinct or more generalized, which is in agreement with the results in Table 1. Moreover, since the outliers may represent abstracts that don’t fit well into any of the topics, fewer outliers in Figure 4 generally suggests that BERTopic is better at capturing the underlying structure of the dataset. In summary, for biomedical applications, particularly those involving detailed and context-sensitive information like opioid-related cardiovascular risks in women, BERTopic offers some advantages over traditional LDA models, and better captures the structure and themes of this dataset, especially when using AI-enhancement.

### The results of the case study: opioids-related cardiovascular risks in women

Both AI-integrated LDA and BERTopic models generated meaningful topics from the case study on opioid-related cardiovascular risks in women, highlighting key areas of concern and clinical relevance. The BERTopic model identified 21 distinct topics, emphasizing specific clinical contexts such as the effects of remifentanil on cardiovascular response during anesthesia, opioid use in different patient populations, and postoperative pain management following cardiovascular surgeries. This model also uncovered themes related to opioid-associated health risks and outcomes in specialized patient groups, including pregnant patients and those undergoing coronary surgery.

In comparison, the LDA model generated 18 topics with a broader focus on the interactions between opioids with cardiovascular and autonomic nervous system responses. This analysis highlighted the association between opioid use and cardiovascular diseases across different populations and detailed the medical complications related to intravenous drug use, such as infective endocarditis and vascular injuries. The LDA model also explored the efficacy and safety of various pharmacological treatments, including opioids, in managing pain and related symptoms, particularly in surgical and postoperative settings.

We will apply the results obtained from the two topic modeling approaches and explore the detailed information for opioid-related cardiovascular risks in women in a future study.

In conclusion, this study demonstrated the effectiveness of both AI-integrated LDA and BERTopic in the text mining of opioid-related cardiovascular risks in women, with BERTopic offering more granular insights, context-specific topics, and a user-friendly working stream through its AI integration. In this study, we did not try to change the traditional/advanced topic modeling algorithms, but to integrate AI tools to enhance or empower the performance of the models. The findings highlighted the importance of AI integration with traditional NLP techniques, which reveal potentially promising directions for future research advancements. By combining the strengths of traditional methods with the advanced pattern recognition and contextual understanding of AI, researchers and developers can build more powerful tools for applications in many fields. As AI continues to evolve, its integration with NLP will likely drive further innovations in how we understand and interact with language. It is reasonable to expect that integrating AI into currently available computational algorithms is a highly promising approach that enhances efficiency, adaptability, and accuracy across various domains. Meanwhile, there exist some challenges to consider. Based on our limited experiences, thoughtful design and validation (such as domain expertise integration) are essential to maximize its benefits.

## Data Availability

Publicly available datasets were analyzed in this study. This data can be found here: https://pubmed.ncbi.nlm.nih.gov/.
