# Supplementary material for: AI-powered topic modeling: comparing LDA and BERTopic in analyzing opioid-related cardiovascular risks in women
Source: Exp Biol Med (Maywood). 2025 Feb 28;250:10389. doi: 10.3389/ebm.2025.10389 (PMC11906279; doi:10.3389/ebm.2025.10389)
Supplement: Supplementary file 1 [file Table1.DOCX]

**Supplemental Table 1a**

| Pmid | Doi | Expert manual review (Y/N) | Title |
| --- | --- | --- | --- |
| 38413849 | 10.1186/s12871-024-02456-2 | Y | Influence of a chronic beta-blocker therapy on perioperative opioid consumption - a post hoc secondary analysis. |
| 37111110 | 10.3390/nu15081891 | Y | Nutritional Status Differs by Prescription Opioid Use among Women of Reproductive Age: NHANES 1999-2018. |
| 37085856 | 10.1186/s12889-023-15629-4 | N | Epidemiology and prevalence of tobacco use in Tehran; a report from the recruitment phase of Tehran cohort study. |
| 35902840 | 10.1186/s12888-022-04135-3 | Y | Diagnostic difficulties in the diagnosis of high acute-phase proteins levels in a teenage drug addicted female patient. |
| 35872529 | 10.1016/j.drugalcdep.2022.109564 | Y | Multiple substance use, inflammation and cardiac stretch in women living with HIV. |
| 35722469 | 10.1155/2022/5095282 | Y | Efficacy of Analgesic Propofol/Esketamine and Propofol/Fentanyl for Painless Induced Abortion: A Randomized Clinical Trial. |
| 34654963 | 10.1007/s00392-021-01945-5 | Y | Drug overdose and the risk of cardiovascular diseases: a nested case-control study. |
| 34619443 | 10.1016/j.drugpo.2021.103463 | Y | Lay knowledge and practices of methamphetamine use to manage opioid-related overdose risks. |
| 34313547 | 10.1080/19490976.2021.1946368 | Y | Chronic opioid use modulates human enteric microbiota and intestinal barrier integrity. |
| 33933061 | 10.1186/s12889-021-10867-w | Y | Opioid use in medical cannabis authorization adult patients from 2013 to 2018: Alberta, Canada. |
| 33896038 | 10.1111/jocs.15570 | Y | STratification risk analysis in OPerative management (STOP score) for drug-induced endocarditis. |
| 33465141 | 10.1371/journal.pone.0245648 | Y | Characterising polypharmacy in the very old: Findings from the Newcastle 85+ Study. |
| 32925320 | 10.1213/ANE.0000000000005005 | Y | Postoperative Critical Events Associated With Obstructive Sleep Apnea: Results From the Society of Anesthesia and Sleep Medicine Obstructive Sleep Apnea Registry. |
| 32790789 | 10.1371/journal.pone.0237359 | Y | Identi&#xfb01;cation and treatment of obstructive sleep apnea by a primary care team with a subset focus on chronic pain management. |
| 31584420 |  | N | Thematic Analysis of Obstetric Anesthesia Cases From the AANA Foundation Closed Claims Database. |
| 31521260 | 10.1016/j.amjcard.2019.07.068 | Y | Frequency of Cardiovascular Events and In-hospital Mortality With Opioid Overdose Hospitalizations. |
| 31502505 | 10.1080/09537104.2019.1665642 | Y | Use of glycoprotein IIb/IIIa antagonists to prevent stent thrombosis in morphine-treated patients with ST-elevation myocardial infarction. |
| 31274241 | 10.1111/hiv.12761 | N | Cause of death among HIV patients in London in 2016. |
| 30849358 | 10.1016/j.ajog.2019.02.056 | Y | Maternal self-harm deaths: an unrecognized and preventable outcome. |
| 30730847 | 10.2478/rjim-2019-0001 | N | The Romanian Society of Internal Medicine's Choosing Wisely Campaign. |
| 30585509 | 10.1080/15563650.2018.1510128 | N | Loperamide misuse to avoid opioid withdrawal and to achieve a euphoric effect: high doses and high risk. |
| 29398161 | 10.1016/j.clinthera.2017.12.011 | Y | Analgesic Use and Risk for Acute Coronary Events in Patients With Osteoarthritis: A Population-based, Nested Case-control Study. |
| 29326869 | 10.1016/j.nicl.2017.12.033 | Y | Prenatal methadone exposure is associated with altered neonatal brain development. |
| 29098610 | 10.1007/s40264-017-0611-5 | Y | Reported Adverse Events with Painkillers: Data Mining of the US Food and Drug Administration Adverse Events Reporting System. |
| 28850356 | 10.1016/j.sjpain.2017.01.006 | Y | Healthcare resource use and costs of opioid-induced constipation among non-cancer and cancer patients on opioid therapy: A nationwide register-based cohort study in Denmark. |
| 27799016 | 10.1176/appi.ps.201600091 | N | Health Promotion for Young Adults With Serious Mental Illness. |
| 27343424 | 10.1016/j.ijcard.2016.06.034 | Y | Opium addiction as an independent risk factor for coronary microvascular dysfunction: A case-control study of 250 consecutive patients with slow-flow angina. |
| 27299617 | 10.1001/jama.2016.7789 | Y | Prescription of Long-Acting Opioids and Mortality in Patients With Chronic Noncancer Pain. |
| 27286724 | 10.1007/s40262-016-0415-2 | Y | Population Genetic-Based Pharmacokinetic Modeling of Methadone and its Relationship with the QTc Interval in Opioid-Dependent Patients. |
| 26940689 | 10.1093/annonc/mdw097 | Y | Are strong opioids equally effective and safe in the treatment of chronic cancer pain? A multicenter randomized phase IV 'real life' trial on the variability of response to opioids. |
| 26939533 | 10.1007/s40268-016-0123-2 | Y | Efficacy and Safety Profile of Diclofenac/Cyclodextrin and Progesterone/Cyclodextrin Formulations: A Review of the Literature Data. |
| 26578201 | 10.1093/eurheartj/ehv567 | Y | Correlates of pre-hospital morphine use in ST-elevation myocardial infarction patients and its association with in-hospital outcomes and long-term mortality: the FAST-MI (French Registry of Acute ST-elevation and non-ST-elevation Myocardial Infarction) programme. |
| 26154713 | 10.1097/DBP.0000000000000188 | N | A Systematic Evaluation of the QTc Interval and Antidepressants in Youth: An Electronic Health Record Study. |
| 25599329 | 10.1001/jamainternmed.2014.6294 | Y | Out-of-hospital mortality among patients receiving methadone for noncancer pain. |
| 24178902 | 10.1007/s13181-013-0352-5 | Y | The Toxicology Investigators Consortium Case Registry--the 2012 experience. |
| 23820570 | 10.1016/j.amjcard.2013.05.037 | Y | QTc interval screening in an opioid treatment program. |
| 23616247 | 10.1007/s40258-013-0031-3 | Y | Cost-utility analysis of duloxetine in osteoarthritis: a US private payer perspective. |
| 23456431 | 10.1007/s12012-013-9204-4 | Y | Impact of opioid pharmacotherapy on arterial stiffness and vascular ageing: cross-sectional and longitudinal studies. |
| 21896615 | 10.1093/eurpub/ckr110 | Y | Psychotropic drugs and accidents in Scania, Sweden. |
| 21333087 | 10.4187/respcare.01014 | Y | Remifentanil improves breathing pattern and reduces inspiratory workload in tachypneic patients. |
| 19902987 | 10.2165/11318070-000000000-00000 | Y | Polymorphism of human cytochrome P450 2D6 and its clinical significance: part II. |
| 16507200 | 10.1017/S0265021506000172 | Y | Vasorelaxant effect of opioid analgesics on the isolated human radial artery. |
| 15868524 | 10.1053/j.jvca.2005.01.027 | Y | Immediate extubation after aortic valve surgery using high thoracic epidural analgesia or opioid-based analgesia. |
| 15610908 | 10.1016/j.arcmed.2004.05.010 | N | Magnetic resonance imaging of severe, long-term, opiate-abuse patients without neurologic symptoms may show enlarged cerebrospinal spaces but no signs of brain pathology of vascular origin. |
| 14709431 | 10.1197/j.aem.2003.08.014 | Y | Out-of-hospital care of critical drug overdoses involving cardiac arrest. |
| 11913483 | 10.1053/euhj.2001.2629 | Y | Vasodilator pre-treatment of human radial arteries; comparison of effects of phenoxybenzamine vs papaverine on norepinephrine-induced contraction in vitro. |
| 9605673 | 10.1097/00000542-199805000-00004 | Y | Profound increase in epinephrine concentration in plasma and cardiovascular stimulation after mu-opioid receptor blockade in opioid-addicted patients during barbiturate-induced anesthesia for acute detoxification. |
| 1363222 | 10.1007/BF03008297 | Y | Anaesthetic management of a parturient with myocardial infarction related to cocaine use. |
| 2225292 | 10.1007/BF03006534 | Y | Alfentanil controls the haemodynamic response during rapid-sequence induction of anaesthesia. |
| 2577251 |  | Y | Continuous high thoracic epidural administration of bupivacaine with sufentanil or nicomorphine for postoperative pain relief after thoracic surgery. |
| 2711995 | 10.1016/0002-9149(89)90186-0 | Y | Infective endocarditis in opiate addicts: analysis of 80 cases studied at necropsy. |
| 3262323 |  | Y | Left ventricular function during propofol and fentanyl anesthesia in patients with coronary artery disease: assessment with a radionuclide approach. |
| 6801952 | 10.1016/0002-9149(82)91967-1 | Y | Large dose sublingual nitroglycerin in acute myocardial infarction: relief of chest pain and reduction of Q wave evolution. |
