# Supplementary figures and images for: AI-powered topic modeling: comparing LDA and BERTopic in analyzing opioid-related cardiovascular risks in women
Source: Exp Biol Med (Maywood). 2025 Feb 28;250:10389. doi: 10.3389/ebm.2025.10389 (PMC11906279; doi:10.3389/ebm.2025.10389)

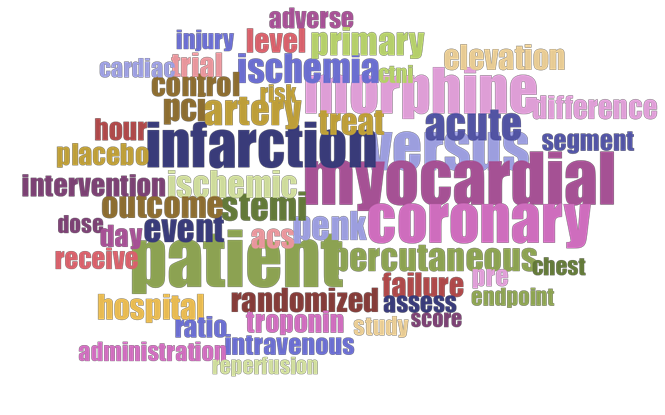

Supplement: Supplementary file 2 [file Image2.TIF]

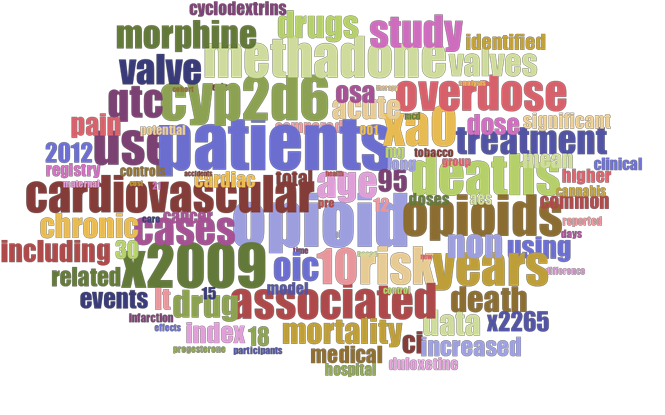

Supplement: Supplementary file 3 [file Image1.TIF]
