# Supplementary material for: AI-powered topic modeling: comparing LDA and BERTopic in analyzing opioid-related cardiovascular risks in women
Source: Exp Biol Med (Maywood). 2025 Feb 28;250:10389. doi: 10.3389/ebm.2025.10389 (PMC11906279; doi:10.3389/ebm.2025.10389)
Supplement: Supplementary file 4 [file Table2.DOCX]

**Supplemental Table 1b**

| Pmid | Doi | Expert manual review (Y/N) | Title |
| --- | --- | --- | --- |
| 38180458 | 10.1097/FJC.0000000000001510 | Y | Central Sleep Apnea in Patients With Coronary Heart Disease Taking P2Y12 Inhibitors. |
| 35714891 | 10.1016/j.jvs.2022.06.004 | Y | Factors associated with lower preoperative quality of life in patients with chronic limb-threatening ischemia in the BEST-CLI trial. |
| 35066983 | 10.1002/ccd.30104 | Y | Sex differences in prehospital analgesia in patients presenting with acute coronary syndromes and their association with clinical outcomes. |
| 34730488 | 10.1532/hsf.4139 | Y | Limb Remote Ischemic Preconditioning Applied During Sevoflurane Anesthesia Does Not Protect the Lungs in Patients Undergoing Adult Heart Valve Surgery. |
| 34706972 | 10.1212/WNL.0000000000012894 | N | Small Fiber Neuropathy Incidence, Prevalence, Longitudinal Impairments, and Disability. |
| 33899498 | 10.1161/JAHA.120.018881 | Y | Impact of Morphine Treatment With and Without Metoclopramide Coadministration on Myocardial and Microvascular Injury in Acute Myocardial Infarction: Insights From the Randomized MonAMI Trial. |
| 33877274 | 10.1001/jama.2021.3414 | Y | Effect of Poloxamer 188 vs Placebo on Painful Vaso-Occlusive Episodes in Children and Adults With Sickle Cell Disease: A Randomized Clinical Trial. |
| 33821671 | 10.1161/JAHA.120.018899 | Y | Troponin, A Predictor of Mortality in Methadone Exposure: An Observational Prospective Study. |
| 33454349 | 10.1016/j.amjcard.2020.12.077 | Y | Comparison of Hospitalization Trends and Outcomes in Acute Myocardial Infarction Patients With Versus Without Opioid Use Disorder. |
| 33432839 | 10.1161/JAHA.120.018762 | Y | Pharmacotherapy in the Management of Anxiety and Pain During Acute Coronary Syndromes and the Risk of Developing Symptoms of Posttraumatic Stress Disorder. |
| 32719075 | 10.1136/openhrt-2020-001307 | Y | Prehospital opioid dose and myocardial injury in patients with ST elevation myocardial infarction. |
| 32543247 | 10.1080/09537104.2020.1779925 | N | Prolonged enoxaparin therapy compared with standard-of-care antithrombotic therapy in opiate-treated patients undergoing primary percutaneous coronary intervention. |
| 32384147 | 10.1182/blood.2019003672 | N | Impact of arginine therapy on mitochondrial function in children with sickle cell disease during vaso-occlusive pain. |
| 31976867 | 10.1016/j.jacc.2019.11.035 | Y | Morphine and Cardiovascular Outcomes Among Patients With Non-ST-Segment Elevation Acute Coronary Syndromes Undergoing Coronary Angiography. |
| 31502505 | 10.1080/09537104.2019.1665642 | Y | Use of glycoprotein IIb/IIIa antagonists to prevent stent thrombosis in morphine-treated patients with ST-elevation myocardial infarction. |
| 31091993 | 10.1161/CIRCHEARTFAILURE.118.005544 | Y | Proenkephalin, an Opioid System Surrogate, as a Novel Comprehensive Renal Marker in Heart Failure. |
| 30850677 | 10.1038/s41598-019-40628-0 | Y | Determinants of high platelet reactivity in patients with acute coronary syndromes treated with ticagrelor. |
| 30767059 | 10.1007/s00392-019-01424-y | Y | Proenkephalin and prognosis in heart failure with preserved ejection fraction: a GREAT network study. |
| 30353444 | 10.1007/s40256-018-0305-0 | Y | Morphine and Ticagrelor Interaction in Primary Percutaneous Coronary Intervention in ST-Segment Elevation Myocardial Infarction: ATLANTIC-Morphine. |
| 30340532 | 10.1186/s12872-018-0936-8 | Y | Intra-coronary morphine versus placebo in the treatment of acute ST-segment elevation myocardial infarction: the MIAMI randomized controlled trial. |
| 29874689 | 10.1055/s-0038-1657768 | Y | Pharmacodynamic Effects of a 6-Hour Regimen of Enoxaparin in Patients Undergoing Primary Percutaneous Coronary Intervention (PENNY PCI Study). |
| 29440010 | 10.1161/JAHA.117.006833 | Y | Effect and Safety of Morphine Use in Acute Anterior ST-Segment Elevation Myocardial Infarction. |
| 29436868 | 10.1080/14017431.2018.1439183 | Y | Effect of oxygen therapy on chest pain in patients with ST elevation myocardial infarction: results from the randomized SOCCER trial. |
| 28373244 | 10.1161/JAHA.116.005426 | Y | Cardioprotective Effects of Intracoronary Morphine in ST-Segment Elevation Myocardial Infarction Patients Undergoing Primary Percutaneous Coronary Intervention: A Prospective, Randomized Trial. |
| 28154162 | 10.1161/JAHA.116.004936 | Y | Outcomes of Physician-Staffed Versus Non-Physician-Staffed Helicopter Transport for ST-Elevation Myocardial Infarction. |
| 28081269 | 10.1371/journal.pone.0170115 | Y | Morphine Does Not Affect Myocardial Salvage in ST-Segment Elevation Myocardial Infarction. |
| 28057251 | 10.1016/j.jacc.2016.10.038 | Y | Proenkephalin, Renal Dysfunction, and&#xa0;Prognosis in Patients With Acute&#xa0;Heart&#xa0;Failure: A GREAT Network Study. |
| 27908843 | 10.2196/jmir.6358 | N | Treatment of Acute Coronary Syndrome by Telemedically Supported Paramedics Compared With Physician-Based Treatment: A Prospective, Interventional, Multicenter Trial. |
| 27734075 | 10.1160/TH16-07-0569 | Y | Efficacy of prasugrel administration immediately after percutaneous coronary intervention in ST-elevation myocardial infarction. |
| 27343424 | 10.1016/j.ijcard.2016.06.034 | Y | Opium addiction as an independent risk factor for coronary microvascular dysfunction: A case-control study of 250 consecutive patients with slow-flow angina. |
| 26626732 | 10.1186/s13049-015-0188-x | Y | Determinants of pre-hospital pharmacological intervention and its association with outcome in acute myocardial infarction. |
| 26578201 | 10.1093/eurheartj/ehv567 | Y | Correlates of pre-hospital morphine use in ST-elevation myocardial infarction patients and its association with in-hospital outcomes and long-term mortality: the FAST-MI (French Registry of Acute ST-elevation and non-ST-elevation Myocardial Infarction) programme. |
| 25725777 | 10.1007/s00392-015-0835-2 | Y | Intravenous morphine administration and reperfusion success in ST-elevation myocardial infarction: insights from cardiac magnetic resonance imaging. |
| 25079300 | 10.1016/j.cardfail.2014.07.008 | Y | Acute heart failure with and without concomitant acute coronary syndromes: patient characteristics, management, and survival. |
| 24140658 | 10.1016/j.jacc.2013.09.037 | Y | Proenkephalin and prognosis after acute myocardial infarction. |
| 22504127 | 10.1253/circj.cj-12-0132 | Y | Cardioprotection from ischemia/reperfusion injury: basic and translational research. |
| 22222469 | 10.1097/ALN.0b013e318242349a | Y | Remote ischemic preconditioning applied during isoflurane inhalation provides no benefit to the myocardium of patients undergoing on-pump coronary artery bypass graft surgery: lack of synergy or evidence of antagonism in cardioprotection? |
| 22055170 | 10.1016/j.amj.2011.08.005 | N | Helicopter scene response for a STEMI patient transported directly to the cardiac catheterization laboratory. |
| 21364138 | 10.1001/jama.2011.235 | Y | Nitric oxide for inhalation in the acute treatment of sickle cell pain crisis: a randomized controlled trial. |
| 20847065 | 10.1510/icvts.2010.243600 | Y | Myocardial injury is decreased by late remote ischaemic preconditioning and aggravated by tramadol in patients undergoing cardiac surgery: a randomised controlled trial. |
| 20555263 | 10.1097/MCA.0b013e32833bdf18 | Y | Coronary stent length predicts PCI-induced cardiac myonecrosis. |
| 18440952 | 10.1093/bja/aen095 | Y | Comparison of S-(+)-ketamine- with sufentanil-based anaesthesia for elective coronary artery bypass graft surgery: effect on troponin T levels. |
| 18078027 |  | Y | Is general anesthesia a risk for myocardium? Effect of anesthesia on myocardial function as assessed by cardiac troponin-i in two different groups (isofluran+N2O inhalation and propofol+fentanyl iv anesthesia). |
| 17467820 | 10.1016/j.ijcard.2007.02.059 | Y | Vaso-vagal reactions during femoral arterial sheath removal after percutaneous coronary intervention and impact on cardiac events. |
| 15713945 | 10.1056/NEJMoa050330 | Y | Complications of the COX-2 inhibitors parecoxib and valdecoxib after cardiac surgery. |
| 10389953 | 10.1097/00006565-199906000-00004 | Y | Effect of ketorolac in pediatric sickle cell vaso-occlusive pain crisis. |
| 10362186 | 10.1016/s0735-1097(99)00095-9 | Y | Effects of naloxone on myocardial ischemic preconditioning in humans. |
| 1401623 | 10.1016/0735-1097(92)90379-2 | Y | Effects of steal-prone anatomy on intraoperative myocardial ischemia. The SPI Research Group. |
| 6801952 | 10.1016/0002-9149(82)91967-1 | Y | Large dose sublingual nitroglycerin in acute myocardial infarction: relief of chest pain and reduction of Q wave evolution. |
